# PD-L1 and PD-L2 expression in the tumor microenvironment including peritumoral tissue in primary central nervous system lymphoma

**DOI:** 10.1186/s12885-020-06755-y

**Published:** 2020-04-05

**Authors:** Motomasa Furuse, Hiroko Kuwabara, Naokado Ikeda, Yasuhiko Hattori, Tomotsugu Ichikawa, Naoki Kagawa, Kenichiro Kikuta, Sho Tamai, Mitsutoshi Nakada, Toshihiko Wakabayashi, Masahiko Wanibuchi, Toshihiko Kuroiwa, Yoshinobu Hirose, Shin-Ichi Miyatake

**Affiliations:** 1 Department of Neurosurgery, Osaka Medical College, 2-7 Daigakumachi, Takatsuki, Takatsuki, Osaka 569-8686 Japan; 2 Department of Pathology, Osaka Medical College, Osaka, Japan; 3 Department of Neurological Surgery, Okayama University, Okayama, Japan; 4 Department of Neurosurgery, Osaka University, Osaka, Japan; 5 Department of Neurosurgery, University of Fukui School of Medical Science, Fukui, Japan; 6 Department of Neurosurgery, Kanazawa University, Kanazawa, Japan; 7 Department of Neurosurgery, Nagoya University, Nagoya, Japan

**Keywords:** Macrophage, PD-L1, PD-L2, Primary central nervous system lymphoma, Tumor microenvironment

## Abstract

**Background:**

The prevalence of programmed death-ligand 1 (PD-L1) and PD-L2 expression on tumor cells and tumor-infiltrating immune cells in primary central nervous system lymphoma (PCNSL) remains unclear. In the present study, we analyzed needle biopsy and craniotomy specimens of patients with PCNSL to compare the PD-L1 and PD-L2 levels in the tumor and surrounding (peritumoral) tissue. We also assessed the correlation between biological factors and the prognostic significance of PD-L1 and PD-L2 expression.

**Methods:**

We retrospectively analyzed the cases of 70 patients histologically diagnosed with PCNSL (diffuse large B-cell lymphoma). Immunohistochemistry for CD20, CD68, PD-L1, and PD-L2 was performed. In cases with specimens taken by craniotomy, the percentages of PD-L1- and PD-L2-positive macrophages were evaluated in both tumor and peritumoral tissue. The Kaplan-Meier method with log-rank test and Cox proportional hazard model were used for survival analysis.

**Results:**

The tumor cells expressed little or no PD-L1 and PD-L2, but macrophages expressed PD-L1 and PD-L2 in most of the patients. The median percentage of PD-L2-positive cells was significantly higher among peritumoral macrophages (32.5%; 95% CI: 0–94.6) than intratumoral macrophages (27.5%; 95% CI: 0–81.1, *p* = 0.0014). There was a significant correlation between the percentages of PD-L2-positive intratumoral macrophages and PD-L2-positive peritumoral macrophages (*p* = 0.0429), with very low coefficient correlation (ρ = 0.098535). PD-L1 expression on macrophages was significantly associated with biological factors (intratumoral macrophages: better KPS, *p* = 0.0008; better MSKCC score, *p* = 0.0103; peritumoral macrophages: low proportion of LDH elevation, *p* = 0.0064) and longer OS (for intratumoral macrophages: high PD-L1 = 60 months, 95% CI = 30–132.6; low PD-L1 = 24 months, 95% CI = 11–48; *p* = 0.032; for peritumoral macrophages: high PD-L1 = 60 months, 95% CI = 30.7–NR; low PD-L1 = 14 months, 95% CI = 3–26). PD-L1 expression on peritumoral macrophages was strongly predictive of a favorable outcome (HR = 0.30, 95% CI = 0.12–0.77, *p* = 0.0129).

**Conclusions:**

Macrophages in intratumoral and peritumoral tissue expressed PD-L1 and PD-L2 at a higher rate than tumor cells. PD-L1 expression, especially on peritumoral macrophages, seems to be an important prognostic factor in PCNSL. Future comprehensive analysis of checkpoint molecules in the tumor microenvironment, including the peritumoral tissue, is warranted.

## Background

Primary central nervous system lymphoma (PCNSL) remains an incurable brain tumor. The standard of care for PCNSL is methotrexate (MTX)-based chemotherapy followed by cranial irradiation. However, there is no reliably effective treatment for recurrent PCNSL after standard-of-care treatment. Nivolumab recently showed survival benefits for recurrent or refractory PCNSL in a small case series and in our case report [1, 2]. Immune checkpoint inhibitors have thus been expected to provide novel treatment for recurrent/refractory PCNSL. A few studies have already reported the programmed death-ligand 1 (PD-L1) and PD-L2 expression on tumor cells and tumor-infiltrating immune cells in PCNSL [3–6], but the expression of these two biomarkers in peritumoral tissue remains unclear.

The use of immune checkpoint inhibitors has provided a major breakthrough in immunotherapy for malignant tumors. Nivolumab has significantly improved the survival of patients with melanoma, non-small cell lung cancer (NSCLC), renal cell carcinoma, and classic Hodgkin lymphoma [7–13]. Biomarkers that predict the treatment response to immune checkpoint inhibitors have also been explored. PD-L1 expression in tumor cells was associated with objective response rates (ORRs) to nivolumab in some studies [9, 14, 15]. However, another study found no difference in the ORRs between PD-L1-positive tumors and PD-L1-negative tumors [16]. Herbst et al. observed PD-L1 staining on tumor-infiltrating immune cells more frequently than on the corresponding tumor cells [17]. Moreover, the association between patients’ responses to anti-PD-L1 treatment with atezolizumab and the expression of PD-L1 on tumor-infiltrating immune cells reached statistical significance in several tumors, whereas the association between the treatment responses and PD-L1 expression on tumor cells did not. Another investigation showed that the survival benefits of atezolizumab were correlated with PD-L1 expression on both tumor cells and tumor-infiltrating immune cells in patients with NSCLC [18]. Finally, in a study on head and neck squamous cell carcinomas, tumors positive for both PD-L1 and PD-L2 had the greatest ORR [19].

Both PD-L1 expression in tumor tissue and that in peritumoral tissue are important for patient prognosis. The presence of PD-L1-positive monocytes in the peritumoral stroma was shown to be an independent prognostic factor of overall survival (OS) in hepatocellular carcinoma (HCC) [20]. Another study of HCC reported that the peritumoral PD-L1 expression in hepatocytes is an independent prognostic factor for survival [21]. Therefore, the expression of checkpoint biomarkers in peritumoral tissue as part of the tumor microenvironment should also be investigated to determine their potential role in the tumor immune escape mechanism. For such investigations, large tumor specimens could be more suitable for the prediction of treatment response to immune checkpoint inhibitors than small tumor samples.

One reason why PD-L1 expression in peritumoral tissue has not been evaluated could be that needle biopsy is often the only tumor tissue collection performed in patients with PCNSL. To fully understand the tumor microenvironment in PCNSL, sampling methods such as craniotomy biopsy might also be used to obtain larger specimens. In the present study, in order to explore the tumor microenvironment in PCNSL, we analyzed the expressions of PD-L1 and PD-L2 in both the tumor and peritumoral tissue. We also analyzed the correlation between survival time and the expressions of PD-L1 and PD-L2.

## Methods

We retrospectively reviewed the cases of 70 patients who were histologically diagnosed with PCNSL (diffuse large B-cell lymphoma) and treated at Osaka Medical College, Nagoya University, Okayama University, Kanazawa University, Osaka University, or Fukui University. All 70 patients underwent surgical resection or biopsy and surgical specimens were taken before the initial treatment (newly-diagnosed PCNSL). We obtained data on the patient characteristics, treatments received, and survival time through chart review, and the formalin-fixed paraffin-embedded tissue samples from the respective institutions. All PCNSLs were diagnosed and classified according to the World Health Organization criteria by pathologists at each institute. The use of materials and clinical data was approved by the institutional ethics committees at Osaka Medical College (Ethics Committee of Osaka Medical College, approval no. 2187) and each participating institute, and was in accord with the Declaration of Helsinki. Informed consent for participation in the study was waived by the ethical committees because this study was a retrospective analysis using archived material, and did not increase risk to the patients.

### Immunohistochemistry and in situ hybridization

For the immunohistochemistry (IHC) analysis, 4-μm-thick sections were cut and the staining was done using an automated staining system (Leica Biosystems, Nussloch, Germany) with antibodies against CD20 (L26; Dako, Santa Clara, CA), CD3 (F7.2.38; Dako), CD68 (KP1; Dako) and PD-L2 (Abcam, Cambridge, MA). For the PD-L1 antibody clone 28–8 (Dako), we used the Dako autostainer Link 48 slide stainer (Code AS480; Dako) following the PD-L1 Dako protocol. For detecting Epstein-Barr virus (EBV), the BOND EBER probe (Leica) was used.

All IHC-stained slides were evaluated and scored by the same board-certified pathologist (H.K.) in a blind fashion. The membranous PD-L1 expression on tumor cells was manually calculated in the most thoroughly stained spot under high magnification. The percentages of PD-L1- and PD-L2-positive tumor cells were calculated by dividing the numbers of PD-L1- or PD-L2-positive tumor cells by the number of all tumor cells, respectively. The percentages of PD-L1- and PD-L2-positive macrophages were calculated in the same manner. In cases with specimens taken by craniotomy, the percentages of PD-L1- and PD-L2-positive macrophages were evaluated in both tumor tissue and peritumoral tissue. Based on the results, the PD-L1 and PD-L2 expressions on tumor cells were categorized into two groups: negative expression (< 1%) and positive expression (≥1%). With regard to macrophages, PD-L1 and PD-L2 expression were categorized into two groups based on the results of a decision tree analysis for survival.

### Statistical analyses

The statistical analyses were performed using JMP® Pro 13.0.0. software (SAS, Cary, NC). Box plots were made using GraphPad Prism ver. 6.03 J software (GraphPad, La Jolla, CA) and showed the median percentage of expression with the 95% confidential interval (CI). Scatter graphs with regression lines were made using JMP software. Comparisons of PD-L1 and PD-L2 expression between groups were conducted using Wilcoxon signed-rank test. Spearman’s rank correlation coefficient was used for determining the correlation between PD-L1 and PD-L2 expressions, and between intratumoral and peritumoral macrophages, respectively. Estimated overall survival (OS) from the date of operation was calculated using the Kaplan-Meier method, and significant differences of OS were determined by log-rank test. Cox proportional hazards model was used to calculate the hazard ratios for risk of death. Probability values < 0.05 were considered significant.

## Results

We evaluated specimens from 70 patients in the analysis. Table 1 shows the patient demographics according to biopsy method. Twenty-eight specimens were taken by needle biopsy, and 42 were taken via craniotomy. Although there were no significant differences in age or Karnofsky performance status (KPS) between the needle biopsy and craniotomy groups, the frequency of cases with a class 3 Memorial Sloan Kettering Cancer Center (MSKCC) score [22] was significantly poorer in the needle biopsy group than the craniotomy group (*p* = 0.0226, Pearson’s chi-square test). The frequency of deep-seated lesions was also significantly greater among patients in the needle biopsy group than those in the craniotomy group (*p* = 0.0163, Pearson’s chi-square test). Not surprisingly, there was a significantly different distribution of the extent of resection between these two groups (*p* <  0.0001, Pearson’s chi-square test). The proportion of patients who were treated with MTX-based chemotherapy was significantly higher in the needle biopsy group than in the craniotomy group (*p* = 0.0078, Pearson’s chi-square test).

**Table 1:** Patient demographics

		Subgroup of biopsy methods		
	All cases (*n* = 70)	Needle biopsy (*n* = 28)	Craniotomy (*n* = 42)	*p* value
Median age (years)	67.5	68.5	67.0	0.8761
Sex, male (%)	38 (54.3)	19 (67.9)	19 (45.2)	0.0627
Median KPS	70	60	80	0.1368
Existence of deep-seated lesion (%)	51 (73.9)	25 (89.3)	26 (51.0)	0.0163
Case with multiple lesions (%)	35 (50.7)	17 (60.7)	18 (43.9)	0.1702
Case with elevated LDH (%)	17 (25.8)	5 (19.2)	12 (30.0)	0.3283
MSKCC score				
Class 1 (age ≤ 50 yrs) (%)	6 (8.8)	3 (11.1)	4 (9.8)	**0.0226**
Class 2 (age > 50 yrs., KPS ≥ 70) (%)	36 (52.9)	9 (33.3)	27 (65.8)	
Class 3 (age > 50 yrs., KPS < 70) (%)	26 (38.2)	15 (55.6)	10 (24.4)	
Extent of resection				
Gross total resection (%)	11 (15.9)	0 (0.0)	11 (26.8)	**< 0.0001**
Partial removal (%)	24 (34.8)	0 (0.0)	24 (58.5)	
Biopsy (%)	34 (49.3)	28 (100.0)	6 (14.6)	
Chemotherapy (%)	60 (87.0)	28 (100.0)	32 (78.1)	**0.0078**
Radiotherapy (%)	48 (69.6)	17 (60.7)	31 (75.6)	0.1867

*KPS* Karnofsky performance status, *MSKCC* Memorial Sloan Kettering Cancer Center

**Fig. 1:**
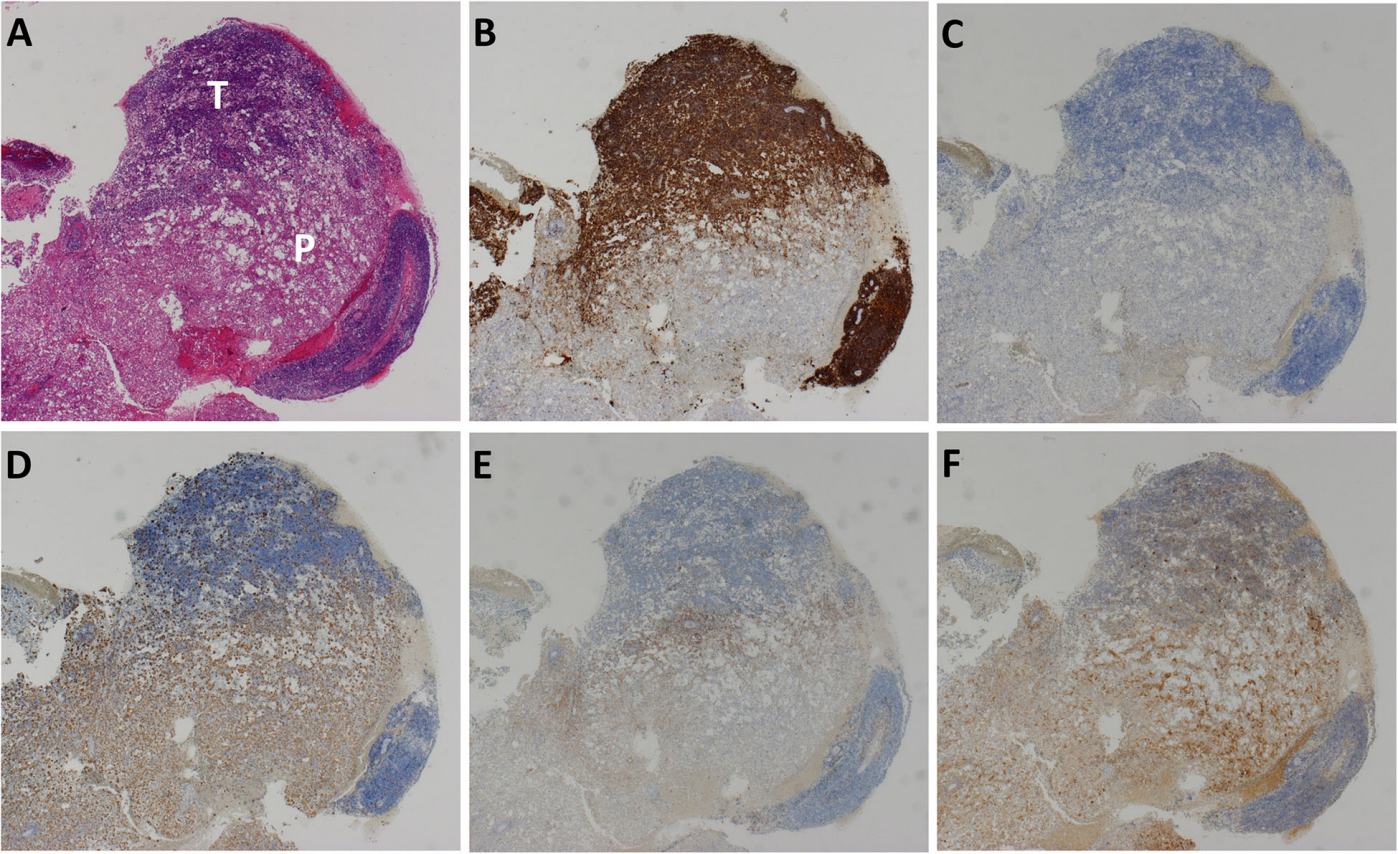
Histopathological microphotographs of tumor and peritumoral tissue. Tumor and peritumoral tissue were stained by hematoxylin and eosin (**a**). For the immunohistochemistry (IHC) analysis, 4-μm-thick sections were cut and the staining was done using an automated staining system (Leica Biosystems, Nussloch, Germany) with antibodies against CD20 (**b**), CD3 (**c**), CD68 (**d**), PD-L1 (**e**), and PD-L2 (**f**) (magnification ×20). P, peritumoral tissue; T, tumor tissue

In all cases, tumor cells were stained by CD20 and not stained by CD3 (Fig. 1a–c). EBV was detected in 10 patients and not detected in the other 60 patients. Generally, tumor cells did not express — but macrophages stained by CD68 did express— PD-L1 in most of the 70 patients with PCNSL (Fig. 1d,e). PD-L2 was frequently expressed on macrophages and was hardly expressed on tumor cells (Fig. 1f).

### PD-L1 and PD-L2 expressions on tumor cells and macrophages in tumor tissue

**Fig. 2:**
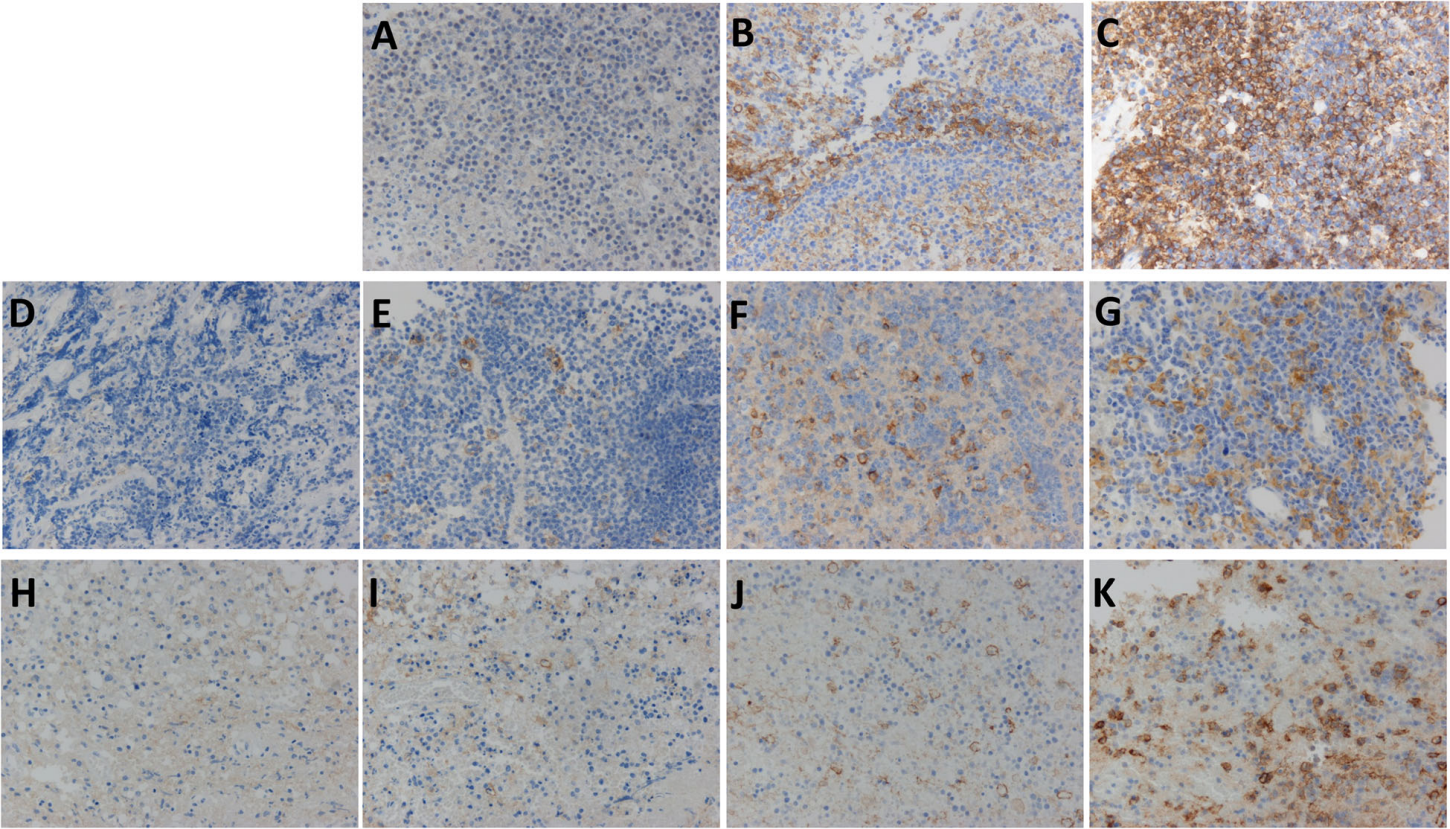
PD-L1 expression on tumor cells and macrophages in tumor tissue and peritumoral tissue. Tumor cells expressed PD-L1 (**a**: none; **b**: moderate; **c**: strong), and macrophages expressed PD-L1 in both tumor tissue (**d**: none; **e**: weak; **f**: moderate; **g**: strong) and peritumoral tissue (**h**: none; **i**: weak; **j**: moderate; **k**: strong) (magnification × 200)

**Fig. 3:**
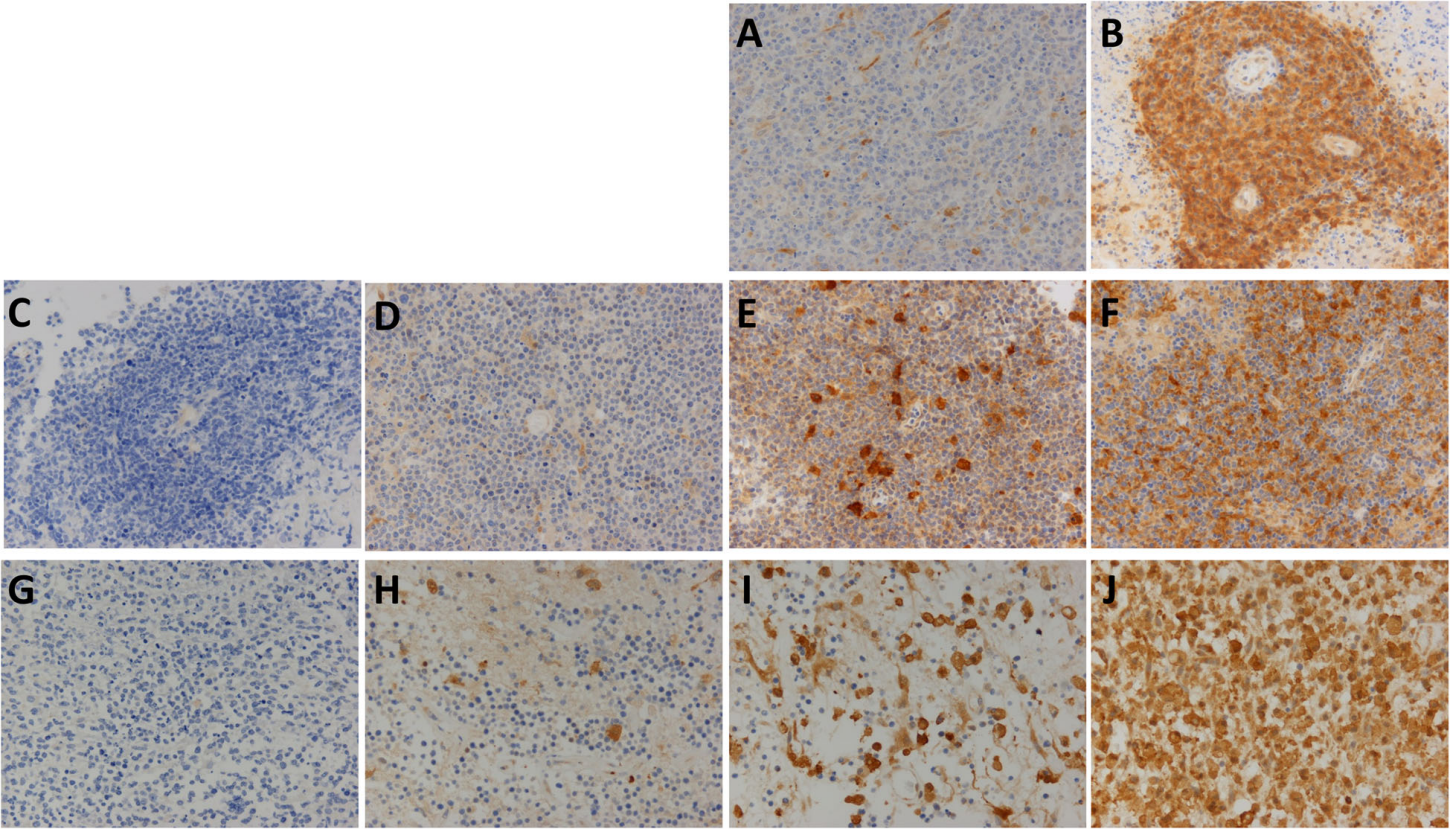
PD-L2 expression on tumor cells and macrophages in tumor tissue and peritumoral tissue. Tumor cells expressed PD-L2 (**a**: none; **b**: strong), and macrophages expressed PD-L2 in both tumor tissue (**c**: none; **d**: weak; **e**: moderate; **f**: strong) and peritumoral tissue (**g**: none; **h**: weak; **i**: moderate; **j**: strong) (magnification × 200)

**Fig. 4:**
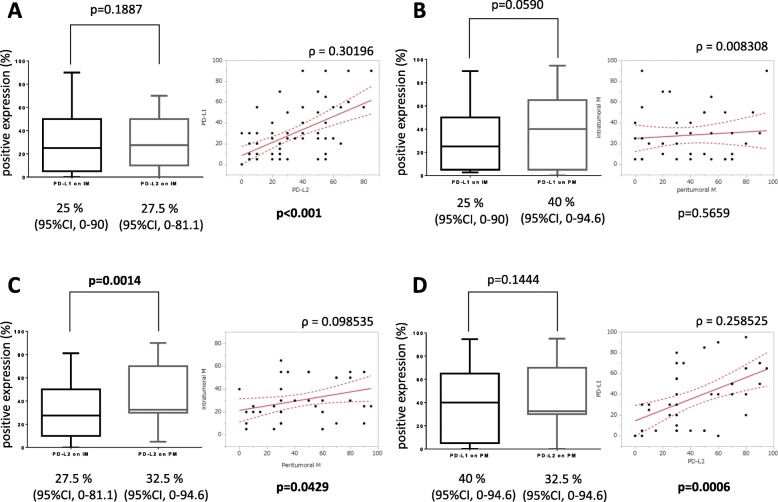
PD-L1 and PD-L2 expression on macrophages in tumor tissue and peritumoral tissue. **a**: PD-L1 and PD-L2 expressions on macrophages in tumor tissue. **b**: PD-L1 expression on macrophages in tumor tissue and peritumoral tissue. **c**: PD-L2 expression on macrophages in tumor tissue and peritumoral tissue. **d**: PD-L1 and PD-L2 expression on macrophages in peritumoral tissue. IM: intratumoral macrophage; PM: peritumoral macrophage. CI, confidential interval

Among the 70 patients, tumor samples from 51 patients showed no PD-LI expression in any of the tumor cells (Table 2, Fig. 2a), while those from 19 patients showed strong or moderate PD-L1 expression in tumor cells (Table 2, Fig. 2b, c). There was no correlation between EBV and PD-L1 expression in tumor cells (*p* = 0.4660, Pearson’s chi-square test). On the other hand, only 2 patients showed no PD-L1 expression on macrophages, with PD-L1 being expressed to varying degrees in the remaining 68 patients (Fig. 2d–g). The median percentage of PD-L1 positive intratumoral macrophages was 25% (95%CI: 0–90). PD-L2 was expressed on tumor cells in only 3 patients (Fig. 3a, b). PD-L2 was expressed in 70, 80 and 95% of tumor cells in these 3 patients. Intratumoral macrophages expressed PD-L2 in the majority of the patients (66 patients) (Fig. 3d–f), and exhibited no PD-L2 expression in only 4 patients (Fig. 3c). The median percentage of PD-L2-positive intratumoral macrophages was 27.5% (95%CI: 0–81.1). In regard to the intratumoral macrophages, there was no significant difference in the percentage of PD-L1-positive and PD-L2-positive macrophages (Fig. 4a, *p* = 0.1887, Wilcoxon signed rank test). There was a significant correlation between the PD-L1 and the PD-L2 expression on macrophages in tumor tissue, but the correlation coefficient was low (Fig. 4a, *p* <  0.001, ρ = 0.30196, Spearman’s rank correlation coefficient).

**Table 2:** Association between biological factors and PD-L1 and PD-L2 expression on tumor cells and macrophages

	Tumor cell			Intratumoral macrophage						Peritumoral macrophage					
	PD-L1			PD-L1			PD-L2			PD-L1			PD-L2		
	Positive (≥1%) (*n*=19)	Negative (<1%) (*n*=51)	*p* value	High (≥20%) (*n*=41)	Low (<20%) (*n*=29)	*p* value	High (≥25%) (*n*=41)	Low (<25%) (*n*=29)	*p* value	High (≥10%) (*n*=31)	Low (<10%) (*n*=11)	*p* value	High (≥70%) (*n*=12)	Low (<70%) (*n*=30)	*p* value
Median age (years)	67	68	0.6724	66	70	0.4036	70	66	0.2829	69	66	0.4063	70.5	66	0.3954
Sex, male (%)	11 (57.9)	27 (52.9)	0.7114	21 (51.2)	17 (58.6)	0.5403	22 (53.7)	16 (55.2)	0.9003	14 (45.2)	5 (45.5)	0.9866	7 (58.3)	12 (40.0)	0.2809
Median KPS	80	70	0.3223	80	60	**0.0008**	70	75	0.9896	80	80	0.8550	80	80	0.4050
Deep-seated lesion (%)	14 (73.7)	37 (74.0)	0.9787	30 (73.2)	21 (75.0)	0.8651	27 (65.9)	24 (85.1)	0.0651	19 (61.3)	7 (70.0)	0.6190	9 (75.0)	17 (58.6)	0.3218
Multiple lesions (%)	8 (42.1)	27 (54.0)	0.3773	21 (51.2)	14 (50.0)	0.9207	22 (53.7)	13 (46.4)	0.5553	13 (41.9)	5 (50.0)	0.6550	6 (50.0)	12 (41.4)	0.6128
LDH elevation (%)	4 (21.1)	13 (27.7)	0.8074	12 (31.6)	5 (17.9)	0.2077	10 (25.0)	7 (26.9)	0.8614	6 (19.4)	6 (66.7)	**0.0064**	2 (16.7)	10 (35.7)	0.2283
MSKCC score															
Class 1 (%)	3 (15.8)	4 (8.2)	0.3861	6 (15.0)	1 (3.6)	**0.0103**	3 (7.3)	4 (14.8)	0.2385	3 (9.7)	1 (10.0)	0.4664	0 (0.0)	4 (13.8)	0.3307
Class 2 (%)	9 (47.4)	27 (55.1)		25 (62.5)	11 (39.3)		25 (61.0)	11 (40.7)		19 (61.3)	8 (80.0)		8 (66.7)	19 (65.5)	
Class 3 (%)	7 (36.8)	18 (36.7)		9 (22.5)	16 (57.1)		13 (31.7)	12 (44.4)		9 (29.0)	1 (10.0)		4 (33.3)	6 (20.7)	

*KPS* Karnofsky performance status, *MSKCC* Memorial Sloan Kettering Cancer Center

### Differences in the PD-L1 and PD-L2 expressions between the intratumoral macrophages and the peritumoral macrophages

In the 42 patients who underwent a craniotomy for their tumor, we compared the expressions of PD-L1 and PD-L2 between the intratumoral and peritumoral tissue. Macrophages in peritumoral tissue expressed PD-L1 in 39 of the 42 patients (Fig. 2h–k). Of the 3 patients who showed negative staining of PD-L1on macrophages in peritumoral tissue, the percentages of PD-L1-positive intratumoral macrophages were 5, 25, and 40%, respectively. The median percentage of PD-L1-positive peritumoral macrophages was 40% (95%CI: 0–94.6) (Fig. 4b). The percentage of PD-L1-positive macrophages tended to be higher in the peritumoral macrophages compared to the intratumoral macrophages, but the difference was not statistically significant (*p* = 0.0590, Wilcoxon signed rank test). There was no correlation between the percentages of PD-L1-positive intratumoral and peritumoral macrophages (*p* = 0.5659, ρ = 0.008303, Spearman’s rank correlation coefficient).

PD-L2 was expressed on peritumoral macrophages in all but 1 of the 70 patients (Fig. 3g–j). The median percentage of PD-L2-positive cells was significantly higher for peritumoral macrophages (32.5%; 95%CI: 0–94.6) than intratumoral macrophages (27.5%; 95%CI: 0–81.1) (Fig. 4c, *p* = 0.0014, Wilcoxon signed rank test). There was significant correlation between the percentages of PD-L2-positive intratumoral and peritumoral macrophages, but the correlation coefficient was very low (Fig. 4c, *p* = 0.0429, ρ = 0.098535, Spearman’s rank correlation coefficient). In the peritumoral macrophages, there was no significant difference between the percentage of PD-L1-positive macrophages and the percentage of PD-L2-positive macrophages (Fig. 4d, *p* = 0.1444, Wilcoxon singed rank test). However, there was a significant correlation between the percentages of PD-L1-positive and PD-L2-positive peritumoral macrophages, although the coefficient of determination was low (*p* = 0.0006, ρ = 0.258525, Spearman’s rank correlation coefficient).

### Association between patient characteristics and PD-L1/PD-L2 expression

None of the patient characteristics were associated with PD-L1 expression on tumor cells (Table 2). Using a decision tree analysis for survival, the PD-L1 and PD-L2 expressions on macrophages were divided into high and low groups. For PD-L1 expression, the cut-off values were 20 and 10% PD-LI-positive intratumoral and peritumoral macrophages, respectively (Table 2). In the case of PD-L2, the cut-off values were 25 and 70% PD-L2-positive intratumoral and peritumoral macrophages, respectively (Table 2). With regard to intratumoral macrophages, the KPS was significantly higher in patients with high expression than in those with low expression of PD-L1 (*p* = 0.0008, Pearson’s chi-square test, Table 2). Patients having a poor MSKCC score were significantly fewer in the high expression group than in the low expression group (*p* = 0.0103, Pearson’s chi-square test, Table 2). In peritumoral macrophages, LDH elevation was significantly more frequent among patients with low expression of PD-L1 than those with high expression of PD-L1 (*p* = 0.0064, Pearson’s chi-square test, Table 2). There was no association between patient variables and PD-L2 expression in either intratumoral or peritumoral macrophages.

### Association between survival time and expression of PD-L1 and PD-L2

**Fig. 5:**
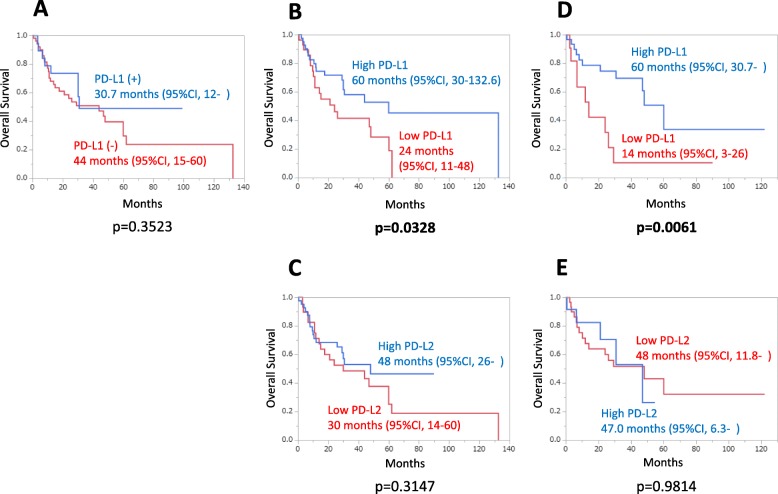
Kaplan-Meier survival curves for overall survival. **a**: PD-L1 expression on tumor cells. **b**: PD-L1 expression on intratumoral macrophages. **c**: PD-L2 expression on intratumoral macrophages. **d**: PD-L1 expression on peritumoral macrophages. **e**: PD-L2 expression on peritumoral macrophages. CI, confidential interval

With regard to PD-L1 expression on tumor cells, the median OS was shorter in patients having tumors with high expression of PD-L1 (30.7 months; 95%CI: 12–not reached) than in patients having tumors with no expression of PD-L1 (44.0 months; 95%CI: 15–60), but the difference was without statistical significance (*p* = 0.3523, Fig. 5a). In relation to intratumoral macrophages, the median OS was significantly longer in the high PD-L1 expression group (60 months; 95%CI: 30–132.6) than in the low PD-L1 expression group (24 months; 95%CI: 11–48) (*p* = 0.0328, Fig. 5b). However, there was no statistical difference in OS between the high and low PD-L2 expression groups (Fig. 5c). Regarding peritumoral macrophages, the median OS was significantly longer in the high PD-L1 group (60 months; 95%CI: 30.7–NR) than in the low PD-L1 expression group (14 months; 95%CI: 3–26) (*p* = 0.0061, Fig. 5d). On the other hand, the median OS was almost the same between the high (47.0 months; 95%CI: 6.3–NR) and low PD-L2 expression groups (48 months; 95%CI: 11.8–NR) (*p* = 0.9814, Fig. 5e). With regard to biological and treatment factors, age > 60 years and elevation of LDH were significantly associated with an increased risk of death (Table 3, age > 60 years; HR = 3.61, 95%CI: 1.40–12.31, *p* = 0.0056; elevation LDH; HR = 2.39, 95%CI: 1.11–4.89, *p* = 0.0265). In addition, PD-L1 expression on intratumoral and peritumoral macrophages and chemotherapy were significantly associated with a decreased risk of death (Table 3, PD-L1 on intratumoral macrophages: HR = 0.50, 95%CI: 0.25–0.96, *p* = 0.0379; PD-L1 on peritumoral macrophages: HR = 0.30, 95%CI: 0.12–0.77, *p* = 0.0129; chemotherapy: HR = 0.28, 95%CI; 0.12–0.76, *p* = 0.0150).

**Table 3:** Cox proportional hazard model for risk of death

	Hazard Ratio (95% CI)	*p* value
Age > 60 years	3.61 (1.40–12.31)	**0.0056**
KPS > 50	0.63 (0.31–1.35)	0.2221
Elevated LDH	2.39 (1.11–4.89)	**0.0265**
Existence of deep-seated lesion	1.04 (0.48–2.12)	0.9076
Existence of multiple lesions	1.05 (0.53–2.04)	0.8976
EB virus-positive	0.95 (0.33–2.24)	0.9199
PD-L1 positive on tumor cells	1.15 (0.55–2.72)	0.7209
PD-L1 positive on intratumoral macrophage	0.50 (0.25–0.96)	**0.0379**
PD-L1 positive on peritumoral macrophage	0.30 (0.12–0.77)	**0.0129**
PD-L2 positive on intratumoral macrophage	0.72 (0.37–1.38)	0.3193
PD-L2 positive on peritumoral macrophage	1.01 (0.39–3.14)	0.9814
Gross total removal (v.s. biopsy)	1.98 (0.76–4.61)	0.1518
Chemotherapy	0.28 (0.12–0.76)	**0.0150**
Radiation therapy	1.73 (0.81–4.12)	0.1593

*KPS* Karnofsky performance status

## Discussion

Our summary of the relevant literature regarding PD-L1 expression in PCNSL is given in Table 4 [3–6]. The rate of PD-L1 expression varied, ranging from 4.1 to 97%. Generally, the rate of PD-L1 expression on tumor cells was lower than that on tumor-infiltrating immune cells. Hayano et al. reported that patients with tumor cells expressing PD-L1 had a significantly longer survival time than patients with tumor cells not expressing PD-L1 [4]; however, there was no significant correlation between the survival time and the PD-L1 expression on tumor stromal cells, although there was a trend for the tumors with PD-L1-negative stromal cells to have longer survival times compared to the tumors with PD-L1-positive stromal cells. Cho et al. also described a correlation between survival and programmed death 1 (PD-1) expression in PCNSLs [5]: the tumors with a high expression of PD-1 had significantly shorter 2-year OS and progression-free survival, but the PD-L1 and PD-L2 expression levels did not correlate with the survival time. The question of which is the most important prognostic biomarker for PCNSL among PD-1, PD-L1, and PD-L2 thus remains unanswered. Moreover, there is no report regarding whether PD-1, PD-L1, and/or PD-L2 is most predictive of the treatment response to an immune checkpoint inhibitor in PCNSL.

**Table 4:** Summary of literatures reporting PD-1, PD-L1, and PD-L2 expression in primary central nervous system lymphoma

	PD-1	PD-L1		PD-L2	
	TIC	TC	TIC	TC	TIC
Berghoff AS, Clin Neuropath 2014	12/20 (60%)	2/20 (10%)	4/20 (20%)		
Hayano A, Anticancer Res 2017		2/48 (4.1%)	25/48 (52%)		
Cho H, Oncotarget 2017	35/76 (46.1%)	54/76 (71.1%)		64/76 (84.2%)	
Sugita Y, Neuropathology 2018					
EBV (+)		12/17 (71%)	16/17 (94%)		
EBV (−)		11/22 (50%)	11/22 (50%)		
This study		19/70 (27.1%)	68/70 (97.1%)	3/70 (4.3%)	66/70 (94.3%)

*TC* tumor cell, *TIC* tumor-infiltrating immune cell including macrophage

In this study, we focused on the expressions of PD-L1 and PD-L2 in peritumoral tissue because we had earlier observed that PD-L1 was markedly expressed on macrophages around tumor tissue in our patient with recurrent PCNSL, who was successfully treated with nivolumab. In that case, tumor cells did not express PD-L1 at all, but tumor-associated macrophages strongly expressed PD-L1, especially in peritumoral tissue [1]. This finding could be a key to solving the mystery of why tumors without PD-L1 expression responded to anti-PD-1 antibody agents. To our knowledge, there has been no published report investigating the expression of checkpoint biomarkers in peritumoral tissue. We thus designed the present study to elucidate the PD-L1 and PD-L2 expressions on macrophages in peritumoral tissue, since PD-L1 and PD-L2 could be important biomarkers in immune checkpoint blockade therapy. Our analyses revealed that the expressions of both PD-L1 and PD-L2 were higher in peritumoral tissue than in tumor tissue, although statistical significance was observed only for PD-L2. Although the PD-L1 expression levels were correlated with the PD-L2 expression levels in both the tumor tissue and the peritumoral tissue, the PD-L1 expression in tumor tissue was not correlated with that in peritumoral tissue. Thus, the expression levels of PD-L1 on macrophages in peritumoral tissue could not be inferred from the corresponding expression levels in tumor tissue.

In our analysis of the association between biological factors and the PD-L1 or PD-L2 expression on tumor cells and macrophages, only PD-L1 expression on macrophages was correlated with better prognostic factors (higher KPS and better MSKCC score in intratumoral macrophages, and lower proportion of LDH elevation in peritumoral macrophages). This association was confirmed by analysis of the correlation between PD-L1 expression and survival time. That is, the only significant association was that patients with high expression of PD-L1 on macrophages had significantly longer OS than those with low PD-L1 expression on macrophages. The hazard ratio of PD-L1 expression on peritumoral macrophages was smaller than that on intratumoral macrophages. Therefore, PD-L1 expression on peritumoral macrophages was a strongly predictive marker for favorable prognosis in PCNSL.

In the initial studies of PD-L1 expression, PD-L1 expression was investigated either in tumor cells or in both tumor and immune cells together without discrimination. A meta-analysis of the correlation between PD-L1 expression and survival in solid tumors showed that overexpression of PD-L1 in tumor tissue was associated with worse OS at both 3 years and 5 years for solid tumors [23]. After that study, it came to be recognized that PD-L1 is expressed not only on tumor cells, but also on tumor-infiltrating immune cells in many cancers. Another meta-analysis revealed that PD-L1 expression on tumor-infiltrating immune cells indicated a decreased risk of death in patients with solid tumors, particularly breast cancer [24]. And yet another study found that PD-L1 expression on tumor-associated macrophages was associated with favorable OS in primary testicular lymphoma [25]. Our present results in patients with PCNSL are consistent with these previous findings that PD-L1 expression on immune cells, including macrophages, was associated with favorable prognosis. In a glioma study in which PD-L1 expression was examined in not only tumor tissue, but also normal brain tissue, there was no expression of PD-L1 in biopsy specimens of the normal brain [26]. Therefore, we believe that macrophages expressing PD-L1 in the tumor and peritumoral tissue could be tumor-associated macrophages. The precise mechanism by which PD-L1 expression is regulated is still unknown. A study using transcriptome analysis suggested that PD-L1 expression on immune cells is regulated through adaptive mechanisms and reflects pre-existing immunity, while PD-L1 expression on tumor cells can be regulated by tumor-intrinsic mechanisms induced by hypoxia [27]. Comprehensive analysis of checkpoint molecules in the tumor microenvironment, including the peritumoral tissue, will be needed to elucidate the tumor immune escape mechanism and pre-existing immune response mechanism to tumors.

A needle biopsy is usually performed in patients in whom PCNSL is preoperatively suspected, because the radical removal of the tumor does not contribute to any improvement of prognosis in patients with PCNSL [28]. Because, in our study, the expression of PD-L1 on peritumoral macrophages predicted a favorable prognosis, and PD-L1 expression on peritumoral macrophages was not correlated with PD-L1 expression on intratumoral macrophages, not only tumor tissue but also peritumoral tissue should be surgically removed and analyzed for PD-L1 expression. It would be optimal to obtain multiple samples including those from peritumoral tissue areas via a needle biopsy. Alternatively, an open biopsy with craniotomy would be one of the surgical options to remove tumor tissue along with peritumoral tissue. A navigation-guided biopsy would be useful to accurately identify multiple targets in tandem with magnetic resonance (MR) imaging [29]. A multimodal image-guided biopsy that is accompanied by MR spectroscopy or MR perfusion imaging may be more accurate for obtaining tissue samples from certain regions [30, 31]. Evers et al. reported that 8 of 11 tumors (73%) showed strong fluorescence of protoporphyrin IX induced by 5-aminolevulinic acid (5-ALA) in their patients with PCNSL [32]. A photodynamic diagnosis of 5-ALA may thus be useful to intraoperatively identify a sample as tumor tissue or peritumoral tissue.

## Conclusions

In the PCNSL patients analyzed in this study, PD-L1 and PD-L2 were expressed on macrophages rather than tumor cells. The PD-L1 expression on macrophages was significantly associated with longer OS. The PD-L1 expression on peritumoral macrophages was strongly predictive of a favorable outcome. The PD-L1 expression on peritumoral macrophages was not correlated with that on intratumoral macrophages. That is to say, the PD-L1 expressions on peritumoral macrophages could not be predicted from the PD-L1 expressions on intratumoral macrophages. Therefore we recommend that peritumoral tissue should be additionally removed via biopsy in patients who are suspected of having PCNSL. To further elucidate the role of PD-L1 expression on macrophages, future comprehensive analysis of checkpoint molecules in the tumor microenvironment, including the peritumoral tissue, is warranted.

## Data Availability

The datasets analyzed during the current study are available from the corresponding author on reasonable request.

## Abbreviations

5-ALA: 5-aminolevulinic acid
CI: Confidential interval
EBV: Epstein-Barr virus
HCC: Hepatocellular carcinoma
IHC: Immunohistochemistry
KPS: Karnofsky performance status
MSKCC: Memorial Sloan Kettering Cancer Center
MR: Magnetic resonance
MTX: Methotrexate
NSCLC: Non-small cell lung cancer
ORR: Objective response rate
OS: Overall survival
PCNSL: Primary central nervous system lymphoma
PD-1: Programmed death 1
PD-L1: Programmed death-ligand 1
